# Unseparated Olive Pruning Waste as a Sustainable Feedstock: DoE-Optimized Extracts with Antioxidant Activity Equivalent to Isolated Leaves

**DOI:** 10.3390/antiox14121441

**Published:** 2025-11-29

**Authors:** Elisabetta Tumminelli, Valeria Cavalloro, Daniela Ratto, Giorgio Marrubini, Emanuela Martino, Paola Rossi, Daniela Rossi, Simona Collina

**Affiliations:** 1 Department of Drug Sciences, University of Pavia, 27100 Pavia, Italy; elisabetta.tumminelli01@universitadipavia.it (E.T.); giorgio.marrubini@unipv.it (G.M.); simona.collina@unipv.it (S.C.); 2 Department of Earth and Environmental Sciences, University of Pavia, 27100 Pavia, Italy; valeria.cavalloro@unipv.it (V.C.); emanuela.martino@unipv.it (E.M.); 3 National Biodiversity Future Centre, Palermo, 90133 Palermo, Italy; 4 Department of Biology and Biotechnology “L. Spallanzani”, University of Pavia, 27100 Pavia, Italy; daniela.ratto@unipv.it (D.R.); paola.rossi@unipv.it (P.R.)

**Keywords:** *Olea europaea*, pruning waste, D-optimal approach, free radical scavenging activity, antioxidant potential, ROS accumulation

## Abstract

Olive cultivation generates substantial pruning waste, yet current valorization strategies focus solely on leaves despite the logistical challenges of separating them. This study optimized the extraction of bioactive compounds from unseparated pruning waste (branches and leaves) using a D-optimal design of experiments to evaluate solvent composition, temperature, and time effects in thirteen experiments. Optimized conditions were scaled up, and extracts were tested via DPPH, ORAC assays, and ROS inhibition in HaCaT cells. Extracts from unseparated pruning waste demonstrated antioxidant capacity equivalent to isolated leaf extracts, and effectiveness in reducing the oxidative stress by approximately 60% compared to the H_2_O_2_ condition. This approach eliminates costly leaf separation while maintaining bioactivity, offering a scalable circular economy solution for olive waste management. The practical implications are substantial: whole pruning waste valorization reduces processing costs and time, decreases agricultural waste by utilizing currently discarded branches, and simplifies supply chains for industries requiring olive-derived antioxidants. Our findings challenge the current paradigm in olive waste biorefining, providing a more economically viable and environmentally sustainable pathway for producing antioxidant-rich extracts suitable for cosmetics, nutraceutical, and pharmaceutical applications.

## 1. Introduction

**Figure 1:**
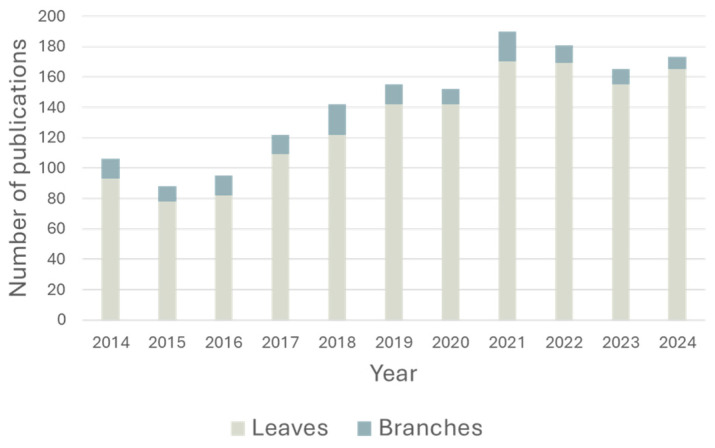
Histogram showing the number of publications per year for olive tree leaves (gray) and branches (light blue). Source: Scopus, search criteria included “*Olea europea*” as the primary term, with filters for leaves and branches.

*Olea europaea* L., a small tree belonging to the Oleaceae family, has been cultivated in the Mediterranean basin since ancient times and has played a significant role in the history, economy, and culture of Western civilization for millennia [1,2,3,4]. In modern times, Spain leads global olive oil production, followed by Greece and Italy, which also represent the states with the highest per capita consumption [2,5]. In Italy, olive cultivation is primarily concentrated in southern regions. However, climate change is reshaping the geographical distribution of olive growing, with northern regions, such as Lombardy, Piedmont, and Veneto, increasingly expanding their cultivation areas. *Olea europaea* L. serves as a sensitive bioindicator of climate change, particularly in terms of temperature, which critically modulates pest interaction [6]. For instance, *Bactrocera oleae* (the olive fruit fly) lays eggs that do not develop below 7.5–10 °C and are not viable above 30–32 °C, while the fungal species *Venturia oleaginea* is especially widespread during hot summers [7,8]. In Lombardy, olive trees find favorable thermal conditions, particularly in winter, in areas around the pre-Alpine lakes and in the Oltrepò Pavese. The latter is a hilly region located in the southern part of Lombardy, just south of the Po River and near the borders with Emilia-Romagna, Liguria, and Piedmont. In the Oltrepò Pavese, although known accessions are few, centuries-old specimens have been identified, testifying to the historical presence of olive cultivation. Several initiatives have been launched to reintroduce and preserve *Olea europaea* L. in this area. Specifically, the OLIMPO project promotes the identification, characterization, and preservation (both in situ and ex situ) of old olive accessions from the region, while the OLIOP project aims to support the development of a local olive oil production chain [9,10]. Beyond these conservation efforts, sustainable management of olive cultivation waste has emerged as a critical challenge [2]. Globally, the olive oil extraction process generates approximately 40 million tons of waste biomass annually, of which more than 20 million tons correspond to dry biomass [11]. In Italy specifically, olive tree cultivation and oil production generate an estimated 5.5–6.5 million tons of residues per year. These include by-products from mills during processing as well as residues from agricultural practices. The first process generates approximately 1.62–1.85 million tons of pomace, 2.24–3.01 million m^3^ of olive mill wastewater, 185–231 thousand tons of branches and leaves, and olive pits representing 10–20% of the weight of fruit. Conversely, residues from agricultural practices are primarily pruning branches and leaves, estimated at 1.65–3.30 million tons per year [12,13,14,15,16]. This substantial volume of waste provides significant opportunities for reuse within a circular economy framework. Among pruning residues, olive tree leaves have been the most extensively studied [17]. Over the past decade (2014–2024), publications on olive leaves have increased significantly, reaching a peak of 165 per year in 2021 (Figure 1). In contrast, branches have received far less attention, with a maximum of only eight publications in 2024 (Figure 1). This disparity in research attention reflects the prevalent assumption that leaves are inherently superior for bioactive extraction, a hypothesis we systematically test in this work.

**Figure 2:**
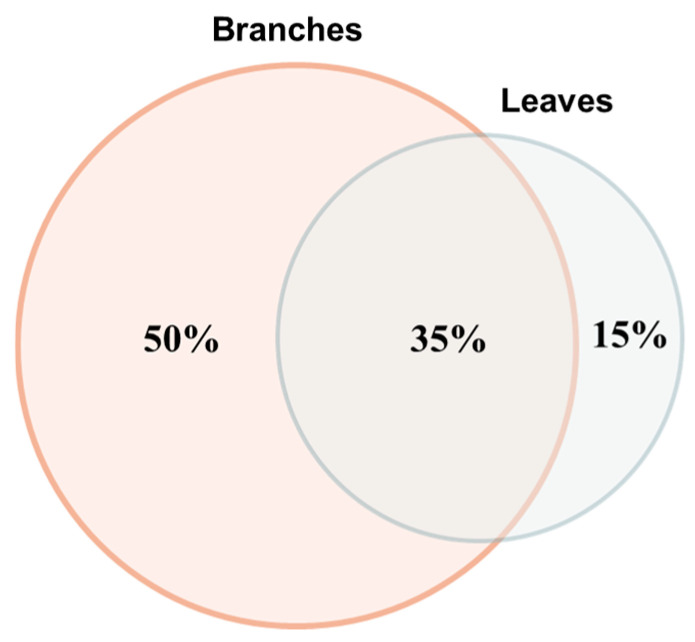
Venn diagram showing the metabolite distribution in *Olea europaea* pruning wastes as percentages of secondary metabolites present exclusively in branches (*n* = 51, 50%), leaves (*n* = 15, 15%), and common to both matrices (*n* = 36, 35%). Total metabolites identified: 102.

The literature data reveal that a total of 102 secondary metabolites have been identified in olive tree leaves and branches, among which 51 exclusively in branches, 15 exclusively in leaves, and 36 that are common to both matrices [18,19,20,21] (Figure 2).

**Figure 3:**
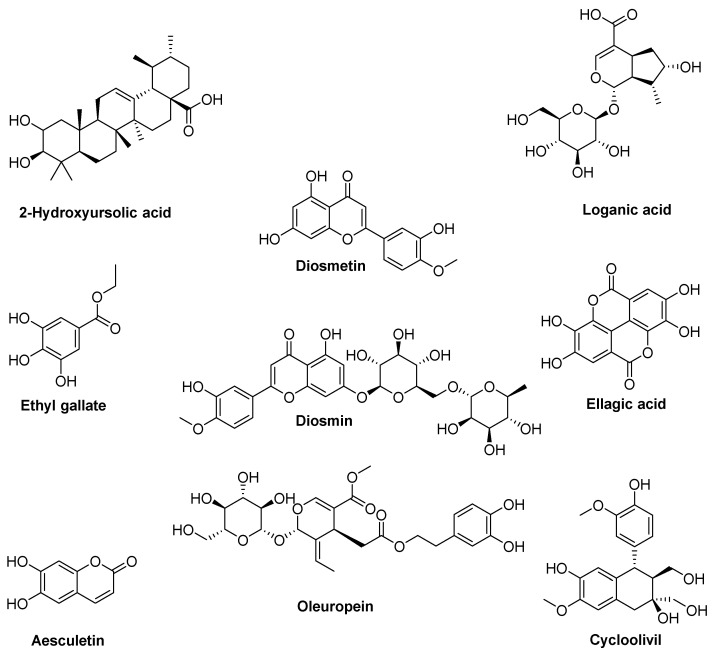
Structure of the most common secondary metabolites present in *Olea europaea* branches and leaves.

Many of these secondary metabolites possess antioxidant potential. Leaf-specific compounds include 2-hydroxyursolic acid, diosmetin, diosmin, ellagic acid, and ethyl gallate loganic acid, while aesculetin and cycloolivil are abundant in branch wood (Figure 3) [22,23,24,25,26,27,28,29,30,31,32,33,34,35,36,37,38]. Among the compounds common to both leaves and branches, oleuropein (Figure 3) stands out as one of the most abundant bioactive molecules and has been extensively studied for its health benefits. This phenolic compound exhibits well-documented antioxidant properties both in vitro and in vivo, attributed to its ortho-diphenolic structure that enables efficient radical scavenging and metal chelation [39]. Evidence demonstrates protective effects against cardiovascular and metabolic diseases, along with good in vivo bioavailability [40]. Moreover, its wound-healing properties have been confirmed in both in vitro and in vivo studies, as described in patent WO2011141611, which investigates oleuropein-based compositions for treating wounds and ulcers in elderly and/or diabetic patients [41,42,43,44,45].

Overall, pruning residues represent a cost-effective and renewable source of biomass for preparing extracts with potential applications in the food, cosmetic, and pharmaceutical sectors. Owing to their antioxidant properties, *Olea europaea* leaf extracts have been proposed as food additives (e.g., for minced poultry or rabbit meat) and as additives for food packaging to preserve quality and extend the shelf life of meat products by slowing oxidation [46]. Various leaf extract-based preparations have also found cosmetic applications: creams for facial face rejuvenation, nanovesicles similar to exosomes formulated in hyaluronic acid/tannic acid hydrogel dressings with dual “defense-repair” effects for treating skin photoaging, oleuropein-based formulations for preventing and treating skin spots (patent EP2218441B1), and anti-inflammatory compositions designed to reduce skin irritation (patent EP1940432B) [47,48,49,50,51]. Additionally, numerous studies have described the antimicrobial, antiviral, anti-inflammatory, antithrombotic, and antihypertensive properties of olive leaf extracts, highlighting their potential pharmaceutical applications [52,53,54,55,56]. Regarding the wood matrix, research has focused mainly on its use as a source of lignin and cellulose for food packaging and biofuel production, as well as a starting biomass for isolating D-mannitol and enolic acid (patent ES2386860A1) [15,57].

Despite extensive research on olive leaf extracts, a critical gap remains: most studies focus exclusively on leaves, requiring costly separation from branches and discarding potentially valuable woody biomass. This study directly addresses this limitation by testing a novel hypothesis: unseparated pruning waste can yield antioxidant extracts equivalent in quality to isolated leaf extracts, thereby eliminating an unnecessary and costly processing step. To test this hypothesis, we developed an extraction protocol for harvesting residues from *Olea europaea* cultivations in the Oltrepò Pavese area, comparing ultrasound-assisted extraction (UAE) and microwave-assisted extraction (MAE) using a systematic design of experiments (DoE) approach. In addition to the free-radical scavenging activity of the extracts, oleuropein was selected as the marker compound to guide extraction optimization, serving as a reliable benchmark for assessing extraction efficiency due to its predominance in *Olea europaea* tissues. We optimized and scaled up the extraction protocol and analyzed the antioxidant potential of the optimized extracts through free radical scavenging assays and in the HaCaT keratinocyte cell line, under both basal and oxidative stress conditions.

## 2. Materials and Methods

### 2.1. Chemicals and Reagents

Formic acid 0.1 M in H_2_O HPLC grade, 2,2-Diphenyl-1-picrylhydrazyl (DPPH), Fluorescein sodium salt, (±)-6-hydroxy-2,5,7,8-tetramethyl-chromane2-carboxylic acid (Trolox), Folin–Ciocalteu’s reagent, gallic acid, 2,2′-Azobis(2-methylpropionamidine) dihydrochloride (AAPH), 3-(4,5-dimethylthiazol-2-yl)-2,5-diphenyltetrazolium bromide (MTT) and 2′,7′-Dichlorodihydrofluorescein diacetate (DCFH–DA), oleuropein, HPLC grade acetonitrile and methanol were supplied by Merck-Sigma-Aldrich (Milan, Italy). Anhydrous sodium carbonate (Na_2_CO_3_) and dichloromethane were acquired from Carlo Erba (Milan, Italy); analytical grade ethanol was supplied by PanReac (Barcelona, Spain). Greenselect^®^ timeline was provided by Indena S.p.A. (Milan, Italy), and 20 cc OESIS HLB was purchased from waters (Milford, MA, USA).

### 2.2. Plant Material

One-year-old lignified shoots of *Olea europaea* L. cultivar Matosso were collected from a 5-year-old plant at the “VALENTINI GERMANO” Ca’ Albertini 2-Frazione Pizzofreddo—Santa Maria della Versa, Pavia. After collection, the plant material was cleaned, cut, and left to dry in a ventilated oven at 35 °C until it reached a constant weight, and then stored in a dark, dry place.

### 2.3. Instruments

Plant material was ground using a Blade-mill (A10 IKA-Werke GmbH & Co., Staufen, Germany). MAE extractions for method setup and optimization were carried out in a microwave mono-mode oven (Discover^®^ Lab-Mate instrument, CEM Corporate, Buckingham, UK) equipped with a power and temperature controller, while for method scale-up a microwave multi-mode oven (MARSX system, CEM Corporate, Buckingham, UK) was employed. UAE was performed using an Elmasonic P 120 H (Elma Schmidbauer GmbH, Singen, Germany). Sample solvent removal was carried out using a Heidolph Laborota 4000 instrument (Heidolph Instruments GmbH & Co., Schwabach, Germany) and a Smart Evaporator C1 (Stepbio, Bologna, Italy). Chromatographic analyses were performed by a Jasco UHPLC system (Jasco Europe, Cremella, Italy) equipped with a quaternary pump model PU-4180, autosampler model AS-4050 with a 5 μL loop, column thermostatic compartment model CO-4065, UV-Vis photodiode array detector (PDA) model MD-4010 with a semi-micro cell, and ChromNAV software (version 2.04.03). ORAC, DPPH, and total phenolic content assays were performed using a FLUOstar^®^ Omega Microplate reader (BMG Labtech, Offenburg, Germany). MTT was carried out by ELx808TM an absorbance microplate reader (Bio-Tek Instruments, Inc., Winooski, VT, USA). DCFH–DA assay was performed using a Leica DM6B WF microscope (Leica microsystems, Milan, Italy), an ORCA-Flash4.0 V3 Digital CMOS camera C13440-20CU (Hamamatsu Photonics, Milan, Italy), and ImageJ software (Version 1.51).

### 2.4. Extraction of Olea europaea Pruning Residues

Two extraction techniques, microwave-assisted extraction (MAE) and ultrasound-assisted extraction (UAE), were employed to prepare extracts from *Olea europaea* L. pruning residues. Two different responses, the total amount of oleuropein (Y_1_) and free radical scavenging activity (% FRS, Y_2_), of the extracts were studied. Y_1_ was computed by HPLC analysis using the external calibration curve method (see Section 2.7) and expressed as mg of metabolite per g of dry matrix weight (DW); Y_2_ was determined through DPPH assay (extract single-dose determination, see Section 2.9.1).

The experimental plan consisted of three continuous factors varying over two levels (X_1_, X_2_, X_3_) and one qualitative factor varying over three levels (the starting biomass). Factor X_1_ was the temperature (studied from 40 to 90 °C for MAE and from 30 to 80 °C for UAE), X_2_ represented the number of extraction cycles (from 1 to 3 cycles), and X_3_ was the solvent composition (ethanol-to-water volume ratio, from 20:80 to 80:20). The sample matrix was the qualitative factor varying from branches (dummy factors coding X_4_ = 1 and X_5_ = 0), to leaves (X_4_ = 0 and X_5_ = 1), to pruning waste (X_4_ = X_5_ = 0), defined as a mixture of branches and leaves without undergoing any separation.

The exhaustive factorial plan thus included a list of 24 experiments (2^3^·3 = 24, Table S1) corresponding to a model equation including one intercept, five linear terms, ten two-term interactions, and eight additional higher-order terms originating from the possible combination of the five factors, considering that the sample matrix can take only three fixed values. Based on preliminary experiments and previous knowledge, the following linear model with interactions was postulated, accounting for nine terms:

$$Y=b_{0}+\sum_{i=1}^{5}b_{i}X_{i}+\sum_{i\neq J=1}^{3}b_{ij}X_{i}X_{j}$$

A D-optimal design was employed to identify the optimal list of experiments, enabling the computation of the coefficients. Three additional independent trials were added to the experimental plan to test the model’s validity (experiments #11, 12, and 13). The experimental plan is described in Tables S2 and S3 for MAE and UAE, respectively.

Specific settings of the MAE and UAE parameters are described below:

- MAE extraction was carried out using a power of 80 Watts for the experiment at 90 °C, 25 Watts for all experiments at 40 °C, and 79 Watts for the experiment at 65 °C; a maximum pressure of 120 PSI; a ramp time of 2 min; and an extraction time of 5 min for each cycle under magnetic stirring.
- UAE extraction was carried out using a working frequency of 37 kHz and an electric power output of 250 W for experiments at 30 °C, 80 kHz and an electric power output of 450 W for experiments at 80 °C, and 55 Hz and 350 W for experiments at 55 °C. Each cycle lasted 10 min.

Other parameters were kept constant for both the extraction methods, including the biomass/solvent ratio at 1/50 g mL^−1^ (i.e., 250 mg of the biomass extracted with 12.5 mL of solvent) and the operator. After each extraction, the mixture was allowed to cool to room temperature and filtered using vacuum filtration with a Büchner funnel. To wash the filter, 3 mL of solvent was applied, varying its composition according to the experimental plan. After filtration, the solvents were removed under reduced pressure (rotary evaporator followed by smart evaporator) until they reached a constant weight.

### 2.5. Chlorophyll Removal After Extraction

Before determining responses Y_1_ and Y_2_, chlorophylls were removed from all the extracts via a solid phase extraction (SPE) method recently developed by us, with slight modifications [58]. Briefly, the cartridges Waters OASIS HLB 1 cc were mounted on the manifold and conditioned with 1 mL of methanol. An amount of 1 mL of each extract previously dissolved in methanol (c = 20 mg mL^−1^) was loaded into cartridges; the vacuum was applied and the cartridge washed with 1 mL of 50% MeOH and 50% water to complete sample elution. At the end the solvent was evaporated under reduced pressure (rotary evaporator followed by smart evaporator) to obtain the purified extracts, while the cartridge was washed with dichloromethane and reactivated with MeOH for future uses.

### 2.6. Extraction Scale-Up

The scalability of the MAE extraction method was evaluated using 6 g of starting biomass. The instrument was set as follows: power of 320 W, maximum pressure of 120 PSI, ramp time of 2 min, extraction time of 5 min, 1 cycle, extraction temperature at 40 °C, 80:20 ethanol/water. The mixture was allowed to cool to room temperature, filtered through vacuum filtration, and finally the solvents were evaporated under reduced pressure (rotary evaporator followed by smart evaporator). Extracts underwent chlorophyll removal prior to HPLC analysis and evaluation of their antioxidant potential.

### 2.7. HPLC-UV/PDA Analysis

HPLC analyses were performed according to Mir-Cerdà et al. using the Kinetex C18 column (4.6 mm ID × 150 mm, particle size 2.6 μm) (Phenomenex^®^, Torrance, CA, USA) [59]. The mobile phase consisted of water containing 0.1% (*v*/*v*) formic acid (A) and acetonitrile (B). The elution was in gradient mode as follows: isocratic elution at 3% B for 3 min, from 3% to 30% of B in 18 min, from 30% to 65% of B in 5 min, up to 90% of B in 2 min. The column was reconditioned by eluting from 90% of B to 3% of B in 3 min, followed by a final isocratic elution at the initial conditions (3% of B) for 7 min. The analyses were performed at 25 °C and at a flow rate of 0.7 mL min^−1^. The injection volume of each sample was 3 μL. The UV–visible absorbance was recorded from 190 to 600 nm, and the quantification of oleuropein was performed at 230 nm applying the external standard method. To this aim a six-points calibration curve, each replicated three times, in the range of 1000—10 μg mL^−1^ was built. Specificity and linearity were evaluated according to the ICH Q2(R2) [60,61]. The quantification limits (QLs) were assessed using the following formulas:

$$QL=5\frac{\sigma }{S}$$

where σ is the standard error of the y-intercept, and *S* is the mean slope of the calibration curve.

The repeatability was verified by performing 10 determinations at the oleuropein test concentration of 0.5 mg ml^−1^.

Extracts, after chlorophyll removal, were dissolved in methanol (1 mg mL^−1^) and filtered through a 0.45 μm GH Polypro membrane (GHP-PerkinElmer, Shanghai China) before HPLC-UV/PDA analysis.

### 2.8. Total Phenolic Content (TPC)

A Folin–Ciocâlteu assay was performed in a multi-well plate according to Everaldo Attard et al. with suitable modifications [62]. Briefly, a stock solution of gallic acid (4.5 nM in methanol) was prepared and diluted in order to build the six-concentrations calibration curve (concentration range 0.125–4.5 mM). Extracts were dissolved in methanol at a concentration of 1 mg mL^−1^. In each well, 100 μL of Folin–Ciocâlteu reagent (2 N in distilled water), 80 μL of sodium carbonate solution (1 M in distillate water), and 10 μL of the sample (extract or gallic acid solution) were added. The plate was incubated at room temperature for 30 min under dark conditions. Absorbance was then measured at 765 nm using a microplate reader. TPC was determined by interpolating the absorbance of the extracts against the gallic acid calibration curve. Results were expressed as mg of gallic acid per g of extract. All tests were performed in triplicate.

### 2.9. Free Radical Scavenging Activity

The free radical scavenging activity of the extracts was evaluated using both DPPH and ORAC assays.

#### 2.9.1. DPPH Assay

Extracts prepared according to the D-optimal experimental plan, after chlorophyll removal, were tested by the DPPH assay using a multi-well plate, according to Brand-Williams et al., with slight modifications [63]. Briefly, for each extract a stock solution was prepared at a concentration of 0.4 mg mL^−1^ in methanol. Then sample, blank, and control solutions were prepared as follows: for sample solution 10 μL of the extract stock solution was mixed with 190 μL of DPPH methanolic solution (0.15 mM); for blank solution 190 µL of methanol was mixed with 10 µL of the extract stock solution; for control solution 190 μL of DPPH methanolic solution (0.15 mM) was mixed with 10 µL of methanol. The plate was incubated in the dark at room temperature for 30 min, and then the absorbance was measured at 515 nm using a microplate reader. The percentage of free radical scavenging activity was calculated using the following formula:

$$\%FRS=100\times \frac{A_{ctrl}-\left(A_{sample}-A_{blank}\right)}{A_{ctrl}}$$

where *%FRS* is the free radical scavenging activity percent, *A_ctrl_* is the absorbance of the control, *A_sample_* is the raw data reading of the sample, and *A_blank_* is the absorbance of the blank.

To determine the IC_50_ value, a stock solution (2.5 mg mL^−1^ in methanol) was prepared for extracts obtained after scale-up (6 g-scale of starting biomass) as well as for Greenselect^®^ and then diluted to generate a six-concentrations curve in the concentration range of 0.05 mg mL^−1^–2.5 mg mL^−1^. % FRS was evaluated as previously described.

#### 2.9.2. ORAC Assay

An ORAC assay was performed in a multi-well plate according to Carvalho et al., with suitable modifications [64]. Briefly, each well was supplemented with 25 μL of a stock solution of the extract (0.1 mg mL^−1^ in methanol) or Trolox (five concentrations ranging from 5.8 mmol to 0.3 mmol in 10 mM phosphate buffer, pH 7.4), and 150 μL of fluorescein solution (10 μM in 10 mM phosphate buffer, pH 7.4) was added. The blank was prepared using 150 μL of fluorescein solution (10 μM in 10 mM phosphate buffer, pH 7.4) added with 25 μL of 10 mM phosphate buffer, pH 7.4.

The plate was incubated at 37 °C for 30 min inside a microplate reader without shaking. Subsequently, 25 μL of AAPH (240 mM in phosphate buffer 10 mM, pH 7.4) was added, and a 120 min kinetic analysis was initiated, with fluorescence readings taken every 20 s (excitation wavelength 485 nm; emission wavelength 520 nm). All tests were performed in triplicate.

The area under the curve (AUC) and net AUC were calculated using the equations reported herein:

$$AUC=\;\frac{1}{R_{1}}\sum_{i=1}^{n}R_{i}$$

where *R*_1_ is the fluorescence reading at the initiation of the reaction, and *R_i_* is the last measurement

The antioxidant activity was determined by calculating the net AUC:

Net AUC=AUC sample−AUC blank

The results were expressed as μmol TE per g of extract by interpolating the net AUC values of the extract against the standard calibration curve of Trolox.

### 2.10. Functional In Vitro Activity

#### 2.10.1. HaCaT Cell Line

The HaCaT cell line, a spontaneously immortalized human keratinocyte line, was obtained from Cell Line Service (Eppelheim, Germany). The cells were maintained in T75 flasks in Dulbecco’s modified Eagle’s medium (DMEM), supplemented with 10% fetal bovine serum (FBS) and 1% penicillin–streptomycin. Culturing was carried out in an incubator at 37 °C with a humidified atmosphere containing 5% CO_2_. The medium was refreshed every 2–3 days, and once the cells reached approximately 90% confluence, they were detached using TrypLE Express and transferred into new flasks with fresh medium. All the reagents were obtained from Gibco Life Technologies (Europe BV, Bleiswijk, The Netherlands).

#### 2.10.2. Sample Preparation

Extracts were dissolved in ethanol/water 80:20 (*v*/*v*) at 75 mg mL^−1^ and then properly diluted in growth medium to reach seven concentrations within the range of 75–1.17 µg mL^−1^.

#### 2.10.3. MTT Assay

The effect of 24 h of treatment with extracts on cell viability was evaluated using the MTT assay, as reported in Desiderio et al. [65]. Briefly, 10,000 cells per well were seeded into a 96-well plate and incubated overnight at 37 °C with 5% CO_2_ to allow adhesion. After 24 h, the culture medium was replaced with fresh medium normal (control condition) or medium supplemented with extract solutions at different concentrations (from 1.17 to 75 µg mL^−1^). After 24 h, 20 µL of MTT (5 mg mL^−1^) was added to each well, and the plate was incubated at 37 °C for 3 h. Next, the formazan crystals were solubilized using 100 µL per well of DMSO. Subsequently, after shaking, the absorbance was acquired at 550 nm using an absorbance microplate reader. All the experiments were conducted in triplicate, and the vehicle ethanol/water 80:20 (*v*/*v*) had no effect on cell viability.

### 2.11. DCFH–DA Assay

The antioxidant effect of extracts was evaluated in the HaCaT cell line with a DCFH–DA assay, as reported in Desiderio et al., 2025 [65]. Briefly, 100,000 cells were seeded on 22 × 22 mm coverslips and incubated overnight at 37 °C with 5% CO_2_ to allow adhesion. After 24 h, the culture medium was changed with fresh medium normal (control condition) or supplemented with the extracts at 75 µg mL^−1^. After 24 h two experimental conditions were created: basal and oxidative stress-induced conditions. For the oxidative stress-induced condition, HaCaT cells were exposed to 500 µM hydrogen peroxide (H_2_O_2_) for 30 min, whereas the basal condition cells were left in fresh medium. Next, cells were incubated with 10 µM DCFH-DA in fresh medium for 30 min at 37 °C in the dark, washed three times in PBS, and then the fluorescence intensity, which directly reflects ROS accumulation levels, was revealed using a microscope at 480 nm excitation and 530 nm emission wavelengths. The images were captured with a camera, and the appropriate software was used to measure the mean optical density (OD) for comparing fluorescence intensity between the different treatments and conditions. All the experiments were conducted at least in triplicate.

### 2.12. Statistical Analysis

All data were analyzed using Microsoft Excel for Windows 365 MSO (Version 18.2002.1101.0), CAT (Chemometric Agile Tool, by Leardi, C. Melzi, G. Polotti), freely downloadable from http://gruppochemiometria.it/index.php/software (accessed on 1 October 2025), developed in R for Microsoft Windows (version 3.2.3, Copyright 2014) [66] and GraphPad Prism 8.0 (GraphPad Software Inc., San Diego, CA, USA). Data are expressed as mean ± standard deviation (SD) of the mean. The Shapiro–Wilk and Bartlett Tests were used to establish the normality of the parameters. Then, population mean value comparisons were carried out using a one-way ANOVA, followed by Bonferroni’s post hoc test for multiple comparisons. Statistical significance was evaluated at a 95% confidence interval, and the differences were considered statistically significant for *p* < 0.05 (*), *p* < 0.01 (**), and *p* < 0.001 (***).

## 3. Results

### 3.1. Setup and Optimization of the Extraction Protocol for Pruning Residues of Olea europaea

**Figure 4:**
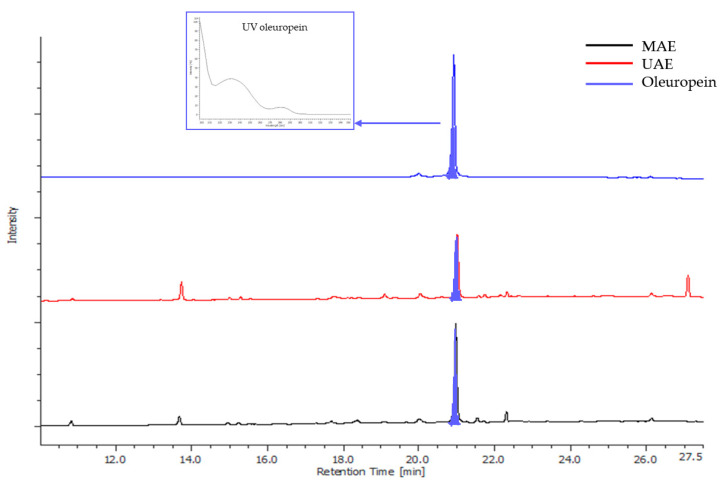
UV trace (λ = 280 nm) of MAE (black) and UAE (red) extracts (80 °C, ethanol/water 50:50 (*v*/*v*), drug/solvent ratio 1/50 (*w*/*v*), extraction times 5 and 10 min for MAE and UAE, respectively) and oleuropein standard (blue). HPLC analyses were performed according to Mir-Cerdà et al. [59].

Based on the literature data, a matrix-to-solvent ratio of 1:50 (*w*/*v*) was selected and constantly maintained throughout the study [67]. Extraction time was preliminarily investigated by comparing the cycle durations of 5 vs. 10 min (MAE) and 10 vs. 20 min (UAE), reflecting the longer times typically required for UAE [68,69,70]. Extractions were performed in triplicate at 80 °C using ethanol/water 50:50 (*v*/*v*). No statistically significant differences in extraction yield or chromatographic fingerprint (peak number and total area) were observed between time points for either method. Consequently, 5 min cycles for MAE and 10 min cycles for UAE were selected to optimize efficiency. HPLC-UV/PDA analysis revealed oleuropein (Rt = 20.93 min) as the main secondary metabolite in all extracts, confirmed by comparison with the authentic standard (Figure 4). The selectivity was ensured by the photodiode array detector.

An experimental design was developed consisting of three continuous factors varying over two levels: temperature X_1_ (40–90 °C for MAE; 30–80 °C for UAE), number of extraction cycles X_2_ (1–3), and ethanol-to-water volume ratio X_3_ (20:80 to 80:20). One qualitative factor with three levels was included to represent the sample matrix: branches, leaves, or unseparated pruning waste containing both branches and leaves (Table S1). A D-optimal design was employed to identify the optimal experimental runs and enable coefficient computation, and three additional independent trials were included to validate the model. The complete experimental designs are described in Tables S2 and S3 for MAE and UAE, respectively. All extracts prepared according to these designs underwent chlorophyll removal prior to HPLC-UV/PDA analysis and DPPH assay to determine responses Y_1_ and Y_2_ (Tables S2 and S3). These responses are discussed separately in the following paragraphs.

Response Y_1_. Oleuropein in pruning residue extracts was quantified using the HPLC-UV/PDA method described by Mir-Cerdà et al. [59]. The method was validated for linearity, quantitation limit (QL), and repeatability [60]. The calibration curve exhibited excellent linearity [y = (4,039,798 ± 75,935.9)x + (124,481 ± 47,244.3); R^2^ = 0.9986] over the concentration range of 10–1000 μg mL^−1^. The QL was established at 60 μg mL^−1^, and method reproducibility was confirmed through ten replicate determinations at 0.5 mg mL^−1^ (RSD = 1.01% for peak area).

Total oleuropein content (Y_1_) is presented in Table S2 (MAE) and Table S3 (UAE). Statistical models for oleuropein recovery using either MAE or UAE were not significant, indicating that oleuropein extraction was robust across the tested factor ranges with no statistically significant differences attributable to the studied variables. Therefore, the mean response values (67.7 ± 18.2 mg g^−1^ DW for MAE and 53.9 ± 4.7 mg g^−1^ DW for UAE) best describe the results. While MAE yielded significantly higher oleuropein recovery than UAE (one-tailed *t*-test for two samples assuming unequal variances, *p*-value 0.0098), it exhibited substantially lower precision, as evidenced by significantly greater variance (F-test for variances, *p*-value 0.00002).

**Figure 5:**
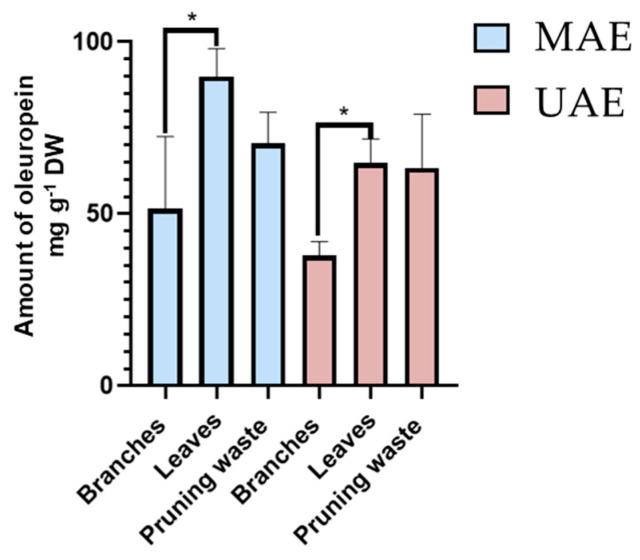
Amount of oleuropein (Y_1_, mg g^−1^ DW by HPLC-UV/PDA) present in MAE (light blue) and UAE (pink) extracts. Results are reported as mean ± SD of experiments 1–4 for branches, experiments 5–6 for leaves, and experiments 7–13 for unseparated pruning waste (Table S2 for MAE and Table S3 for UAE). *p*-values * *p* < 0.05.

To further elucidate the influence of biomass type on Y_1_, data were analyzed by biomass category: branches (experiments 1–4), leaves (experiments 5–6), and unseparated pruning waste (experiments 7–13) (Tables S2 and S3 for MAE and UAE, respectively). By isolating matrix effects from process factor variability, one-way ANOVA revealed that branches contained significantly less oleuropein than leaves, which yielded comparable amounts of this metabolite to unseparated pruning waste (95% confidence level). These findings were consistent for both MAE and UAE methodologies (Figure 5).

Response Y_2_. After evaluating the effect of continuous and qualitative factors on oleuropein content (Y_1_), the free radical scavenging activity of the extracts (% FRS, Y_2_) was examined. The results are reported in Tables S2 and S3 for MAE and UAE, respectively. Notably, none of the models computed for % FRS using either MAE or UAE procedures were statistically significant. This indicates that % FRS values remain robust regardless of changes in the evaluated factors. Moreover, no significant differences were observed between the mean % FRS values obtained with MAE (56.6 ± 9.0%) and UAE (55.7 ± 16.9%), with variances that were not significantly different (F-test for variances, *p* value = 0.02).

**Figure 6:**
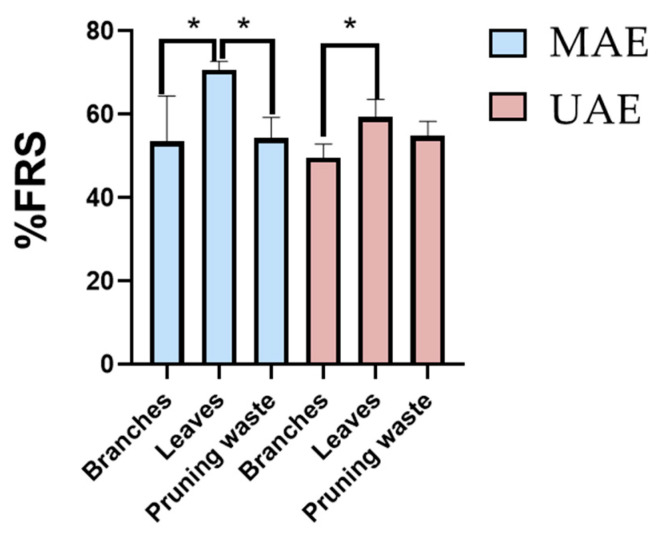
Free radical scavenging activity percentage (% FRS) by DPPH assay for MAE (light blue) and UAE (pink) extracts. Results are expressed as mean ± SD of experiments 1–4 for branches, experiments 5–6 for leaves, and experiments 7–13 for unseparated pruning waste (Table S2 for MAE and Table S3 for UAE). *p*-values * *p* < 0.05.

Lastly, as performed for oleuropein content (Y_1_), the effect of matrix type on the percentage of FRS was assessed by removing the variability introduced by process factors. One-way ANOVA revealed that matrix type has a significant effect on the response (95% confidence level). Specifically, leaves exhibited significantly higher FRS activity than branches, while showing comparable (UAE) or slightly higher (MAE) antioxidant potential compared to pruning waste (Figure 6).

Taken together, the results reported pointed out that MAE is the best extraction method, (i) giving better results associated with oleuropein extraction yield and % FRS comparable to that of UAE, and (ii) requiring half the extraction time of UAE. Experimental conditions involving an ethanol/water 80:20 (*v*/*v*) extraction solvent, one 5 min microwave cycle at 40 °C with a power of 25 W, and a 1:50 plant material-to-solvent ratio were identified as optimal for the biomasses considered in the study and were thus properly scaled up.

### 3.2. Evaluation of Extraction Method Scalability

**Figure 7:**
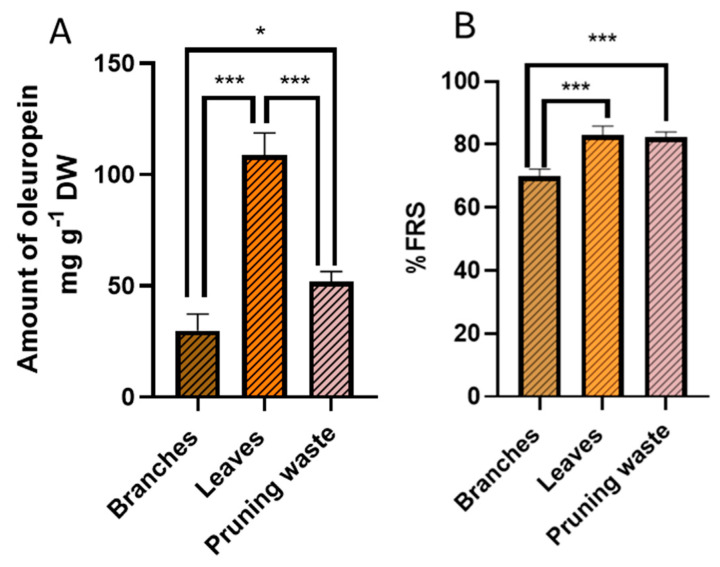
(**A**) amount of oleuropein (mg g^−1^ DW) by HPLC-UV/PDA and (**B**) free radical scavenging activity percentage (% FRS) by DPPH assay for g-scale extracts. Results are reported as mean ± SD of three experiments. *p*-values * *p* < 0.05; *** *p* < 0.001.

Having identified MAE as the superior extraction technique, delivering higher oleuropein yields with comparable % FRS to UAE while requiring half the extraction time, the optimized conditions were scaled up to assess industrial feasibility. The optimal parameters (ethanol/water 80:20 (*v*/*v*), single 5 min microwave cycle at 40 °C, 1:50 solid-to-solvent ratio) were applied to approximately 6 g of each matrix (branches, leaves, and pruning waste) in triplicate using a multimodal microwave oven, as detailed in the experimental section. For comparative purposes, 0.25 g of each matrix were also extracted in triplicate following the optimized conditions, operating on the same batch used for the 6 g-scale extraction. Notably, comparable extraction efficiency was achieved at both 0.25 g and 6 g scales (24-fold scale-up), as either extraction yield (19.7 ± 1.7% vs. 21.5 ± 1.7% for branches, 40.0 ± 0.4%vs 43.2 ± 1.3% for leaves, 25.1 ± 2.9% vs. 27.0 ± 1.1% for pruning waste) or oleuropein recovery (37.1 ± 4.5 vs. 29.8 ± 7.4 mg g^−1^ DW for branches, 96.4 ± 11.6 vs. 108.6 ± 10.0 mg g^−1^ DW for leaves, 47.6 ± 9.5 vs. 51.7 ± 4.7 mg g^−1^ DW for pruning waste) was statistically unvaried within the same biomass (two-tails *t*-test). Similarly, FRS potential also remained statistically equivalent between scales (76.5 ± 5.5% vs. 69.9 ± 2.5% for branches, 84.0 ± 5.5% vs. 82.9 ± 2.8% for leaves, 92.2 ± 6.0% vs. 82.3 ± 1.7% for pruning waste, two-tails *t*-test), thus demonstrating an excellent reproducibility of the scale-up process for all the biomass considered. Overall, based on the results reported above, none of the biomass considered exhibited scale-dependent behavior. Focusing on the larger 6 g-scale, one-way ANOVA evidenced that, although leaves are the highest yielding biomass concerning oleuropein extraction, with superior results to both branches and pruning waste (95% confidence level, Figure 7 panel A), unseparated pruning waste is equal in performance to leaves in terms of FRS potential and it is superior to branches (*p* < 0.001, Figure 7 panel B). Then, extracts prepared from 6 g of feedstock (branches, leaves, and pruning waste), after chlorophyll removal, were subjected to a comprehensive characterization of their antioxidant potential through DPPH, ORAC, and DCFH-DA assays, as described in the following paragraphs.

### 3.3. Total Phenolic Content (TPC) and Antioxidant Potential of the Extracts

**Figure 8:**
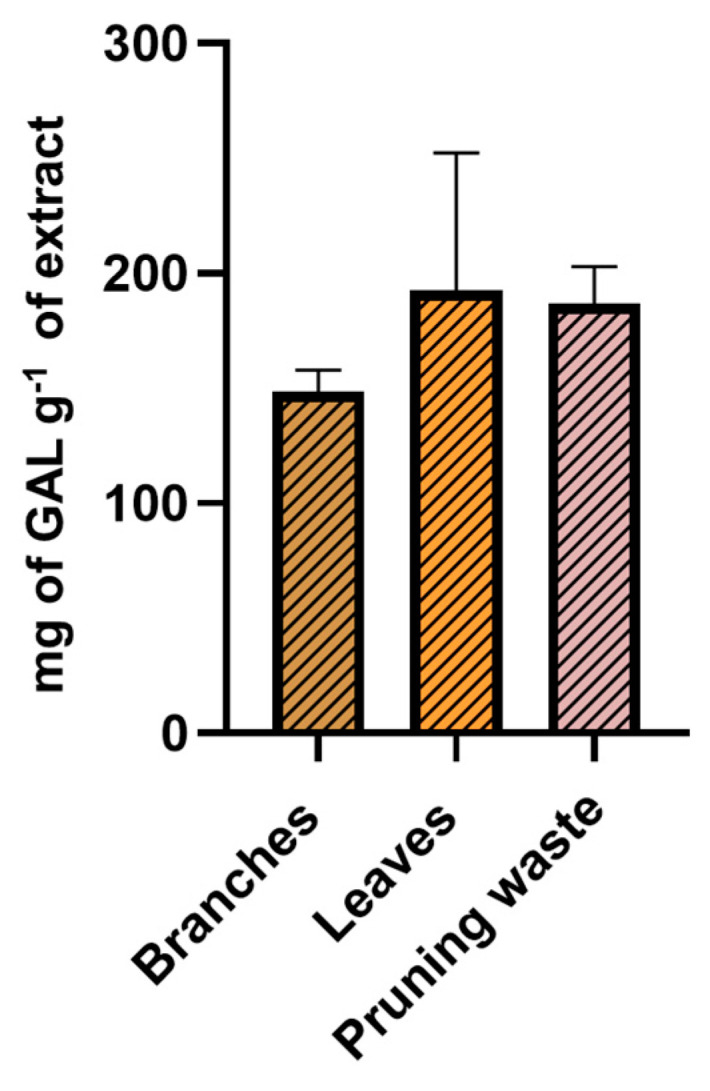
TPC of g-scale extracts from leaves, branches, and pruning waste determined through Folin–Ciocâlteu assay. Results are reported as mean ± SD of three experiments.

Before evaluating the antioxidant potential of leaf-, branch-, and pruning waste-derived extracts prepared during scale-up, the Folin–Ciocâlteu assay was carried out to determine their total phenolic content (TPC). Analyses were conducted in triplicate, and results were expressed as mg of gallic acid per g of extract [gallic acid calibration curve Y = (4.22 ± 0.06)x − (0.011 ± 0.007), R^2^ = 0.9995]. Of note, no significant differences in the TPC of extracts from leaves, branches, and the pruning waste emerged from data analysis (one-way ANOVA, Figure 8, Table S4).

### 3.4. Evaluation of Free Radical Scavenging Activity of the Extracts

**Figure 9:**
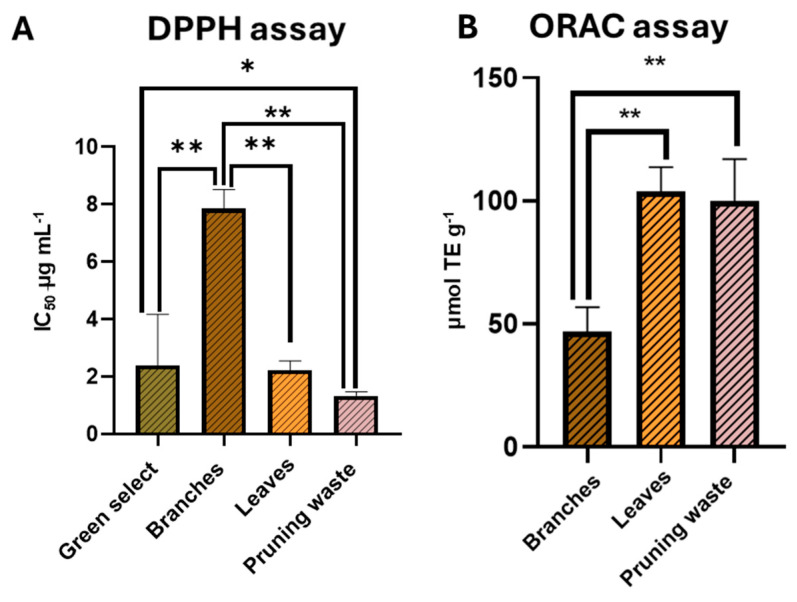
(**A**) IC_50_ values by DPPH assay for g-scale extracts and Greenselect^®^ and (**B**) mmol equivalents of Trolox per g of g-scale extracts through ORAC assay. Results are reported as mean ± SD of three experiments. *p*-values * *p* < 0.05; ** *p* < 0.01.

Following the evaluation of the scalability of the extraction method, the obtained extracts were used to determine the IC_50_ values using the DPPH assay through dose–response curves (experiments performed in triplicate). GreenSelect^®^ was employed as a reference antioxidant agent for comparative purposes due to its well-established antioxidant properties (Figure S1) [71]. Notably, no significant difference was observed between the IC_50_ values of leaf and pruning waste extracts (2.2 ± 0.3 µg mL^−1^ and 1.3 ± 0.1 µg mL^−1^, respectively), whose antioxidant activities were comparable and significantly higher than that of branch extract (IC_50_ = 7.9 ± 0.7 µg mL^−1^, *p* < 0.01, one-way ANOVA, Figure 9 panel A). Moreover, pruning waste extract was the only sample demonstrating antioxidant potential significantly higher than GreenSelect^®^ (IC_50_ = 2.3 ± 1.7 µg mL^−1^, *p* < 0.05, one-way ANOVA, Figure 9 panel A). To corroborate these findings, the ORAC method was employed to further investigate the free radical scavenging potential of the extracts through single-dose determination (experiments performed in triplicate). The results are expressed as μmol Trolox equivalents (TE) per g of extract [Trolox calibration curve: y = (2.6383 ± 0.27)x + (2.5454 ± 0.8), R^2^ = 0.97]. Consistent with the DPPH results, the antioxidant potential was significantly affected by the starting biomass. Once again, leaf- and pruning waste-derived extracts showed comparable antioxidant potential, both being significantly higher than branch extract (*p* < 0.01, one-way ANOVA, Figure 9 panel B, Table S5).

### 3.5. Evaluation of Antioxidant Effect in HaCaT Cell Line

The final step of this project involves testing extracts from branches, leaves, and pruning waste biomasses on the HaCat cell line to further corroborate their antioxidant potential.

**Figure 10:**
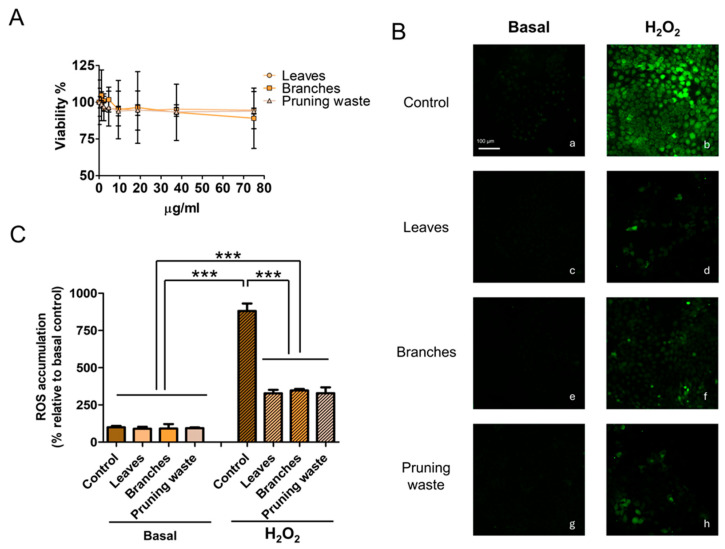
(**A**) Effect of leaf-, branch-, and pruning waste-derived extract treatment for 24 h on HaCaT cell viability. (**B**) Representative images of intracellular ROS accumulation stained with DCFH-DA in HaCaT cells in different experimental conditions. The left panels represent the basal condition (**a**: control; **c**: leaves; **e**: branches; **g**: pruning waste). The right panels show the H_2_O_2_-induced oxidative stress condition (**b**: control, **d**: leaves; **f**: branches; **h**: pruning waste). The scale bar denotes 100 μm. (**C**) ROS accumulation quantification in percentage relative to basal control condition in HaCaT cells. Results are reported as mean ± SD of at least three experiments. *p*-values *** *p* < 0.001.

An MTT assay was first conducted, evidencing a cell viability of approximately 90% after 24 h of treatment at the highest tested concentration (75 µg mL^−1^) for all the extracts, thus demonstrating a good cytocompatibility (Figure 9 panel A). To evaluate the antioxidant effect of the extracts, the DCFH-DA assay was then carried out in both basal and H_2_O_2_-induced oxidative stress conditions. As reported in Figure 10 panels B and C, 24 h of treatment with the extracts at 75 µg mL^−1^ concentrations did not significantly change the ROS accumulation in HaCaT cells (ROS accumulation % relative to the control in leaves sample: 89.30 ± 40.43; branches sample: 90.84 ± 30.42; pruning waste sample: 93.59 ± 15.30). Next, we evaluated the ability of the extracts to prevent oxidative stress. As shown in Figure 10 panels B and C, the 30 min treatment with 500 µM H_2_O_2_ significantly increased the production of ROS (ROS accumulation%: 880.91 ± 158.32; *p* < 0.001, one-way ANOVA) in HaCaT cells. Notably, 24 h of pretreatment with leaf- (ROS accumulation%: 328.33 ± 71.68), branch- (ROS accumulation%: 347.05 ± 28.55), and pruning waste- (ROS accumulation%: 329.07 ± 100.86) derived extracts at 75 µg mL^−1^ significantly prevents the ROS accumulation induced by 30 min 500 µM H_2_O_2_ treatment in HaCaT cells, reducing the oxidative stress by approximately 60% compared to the H_2_O_2_ condition (Figure 10, for all samples *p* < 0.001, one-way ANOVA). The confidence levels corresponding to the 95% confidence intervals for all experimental groups are reported in Table S6.

## 4. Discussion

*Olea europaea* cultivation is expanding worldwide due to increasing demand for olive-based food products. Among the various biomasses derived from this species, leaf extracts have been the most extensively studied, primarily due to their antioxidant potential. However, olive tree pruning generates substantial quantities of residual biomasses that remain underutilized. The present work aimed to evaluate whether different pruning biomasses (branches, leaves, or their unprocessed mixture, i.e., pruning waste) affect the antioxidant potential and oleuropein content of the resulting extracts. To achieve this goal, a design of experiments (DoE) approach was employed to optimize extraction conditions that maximize both antioxidant activity and oleuropein yield, a key bioactive compound produced by *Olea europaea* with antioxidant properties. Specifically, microwave-assisted extraction (MAE) and ultrasound-assisted extraction (UAE) protocols were investigated, as both techniques enhance extraction efficiency while reducing time and energy consumption compared to traditional methodologies.

The continuous factors considered in the experimental plan are temperature, solvent composition, and number of cycles, and they have been selected to shed light on divergent or omitted data reported in the literature. Specifically, already published MAE and UAE protocols used for the extraction of *Olea europaea* pruning residues (mainly leaves) have been described as optimized using extraction mixtures mainly composed of ethanol in water in different compositions, within a wide range of temperature (from room temperature to 86 °C) [69,72]. In this work, two different temperature ranges were tested to evaluate the full potential of both techniques: 40–90 °C for MAE and 30–80 °C for UAE. MAE enables extraction above the solvent boiling point due to pressurized conditions, while UAE achieves efficient extraction at lower temperatures through cavitation effects. Furthermore, to the best of our knowledge, the number of extraction cycles has never been investigated. Regarding qualitative factors, the starting biomasses have never been compared in the same experimental plan, and an unprocessed pruning mixture has never been investigated as a valuable alternative to leaves.

Considering the high number of factors (three continuous factors and two dummy factors for the starting biomass), a D-optimal experimental plan was adopted to identify the optimal list of experiments, enabling the computation of the coefficients for the model linear equation. Extracts prepared according to the D-optimal experimental plan were treated to remove chlorophylls. These green pigments may hinder both analytical procedures and fluorometric assays. Therefore, after chlorophyll removal, extracts were tested using the DPPH assay at a single concentration to evaluate their FRS potential. Furthermore, oleuropein content was quantified using a calibration curve developed by applying an HPLC-UV/PDA method previously validated according to the ICH Q2(R2) and USFDA guidelines. The results demonstrated that the continuous factors selected in this study did not significantly affect the responses. Independent experiments performed in triplicate with each biomass further validate that the two responses were robust in the experimental domain considered and that they are only correlated with the starting biomass. Of note, MAE yielded a higher oleuropein content compared to UAE, despite requiring only half the cycle time. Consequently, MAE was selected for scale-up, and extraction conditions were optimized to minimize time, energy consumption, and environmental impact (one cycle, 40 °C, ethanol/water 80:20).

The method was successfully scaled from milligram to gram quantities, producing extracts with total extraction yields, free radical scavenging percentages (% FRS), and oleuropein contents consistent with those obtained at a smaller scale. The MAE methodology shows potential for industrial implementation. To further characterize the extracts, the Folin–Ciocâlteu assay was performed, revealing no significant differences in total phenolic content among the three biomass matrices. The free radical scavenging properties were better investigated by determining IC_50_ values through the DPPH assay and by ORAC assays (single-dose determination) [73,74]. These complementary methods differ in specificity: the DPPH assay is rapid and measures direct radical scavenging, while the ORAC assay involves 120 min of kinetic measurement and evaluates antioxidant capacity against peroxyl radical-induced oxidation. One-way ANOVA revealed statistically significant differences in DPPH IC_50_ and ORAC values between leaf and branch extracts. Importantly, no statistically significant differences were observed between leaf and pruning waste extracts, suggesting that unprocessed pruning waste represents a valuable alternative to isolated leaves.

Lastly, extracts from leaves, branches, and pruning waste were evaluated in HaCaT keratinocytes to assess their potential for topical cosmetic applications. Since keratinocytes constitute the primary cellular component of the epidermis, the main target of cosmetic treatments, this cellular model provides relevant insights into skin compatibility and efficacy [75].

**Figure 11:**
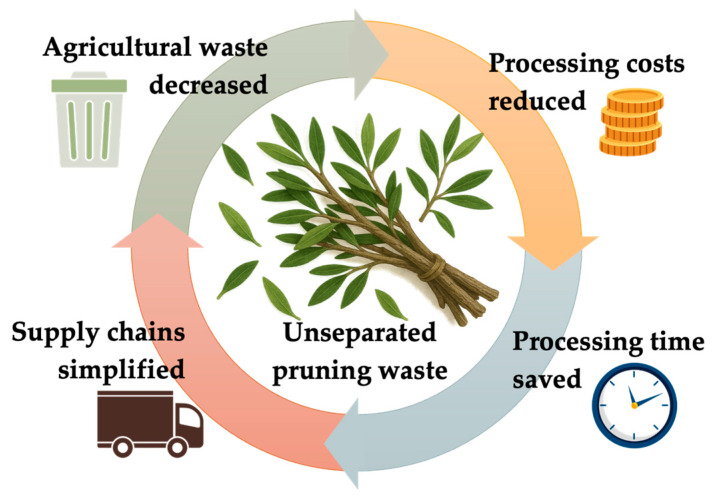
Benefits related to the use of unseparated pruning residues for preparing extracts endowed with antioxidant properties.

Following confirmation of cytocompatibility through MTT assay, the antioxidant capacity of extracts was assessed using the DCFH-DA assay under both basal and oxidative stress conditions. Under basal conditions, no significant reduction in ROS was observed. This is an expected result given that physiologically healthy HaCaT cells maintain inherently low baseline ROS levels [76]. However, upon exposure to H_2_O_2_-induced oxidative stress, all extracts demonstrated strong protective effects. Critically, branch and unseparated pruning waste extracts exhibited antioxidant activity statistically equivalent to leaf extract, challenging the prevailing assumption that leaves must be isolated to obtain high-quality antioxidant extracts from olive pruning biomass [77]. This finding represents a paradigm shift for olive pruning waste valorization with clear benefits: the standard practice of separating leaves from branches, a time-consuming and costly step, appears unnecessary for producing extracts with comparable bioactivity, thus offering a scalable circular economy solution for olive waste management (Figure 11).

## 5. Conclusions

This study demonstrates that unseparated olive pruning waste yields extracts with antioxidant properties equivalent to those from isolated leaves, thereby eliminating the need for leaf–branch separation, a labor-intensive step universally applied in previous valorization studies. By systematically comparing three biomass matrices (branches, leaves, and unprocessed pruning waste) using DoE-optimized extraction protocols, we provide the first evidence that whole pruning waste represents a superior feedstock for antioxidant extract production compared to isolated leaves. The practical implications are substantial: shifting from leaf isolation to whole pruning waste valorization would (1) reduce processing costs and time, (2) decrease agricultural waste by utilizing branches currently discarded or burned (when still allowed), and (3) simplify supply chains for industries requiring olive-derived antioxidants. However, the variable leaf-to-branch ratio in unprocessed pruning waste may affect batch-to-batch reproducibility—a concern readily addressed through small-scale quality assessment before industrial scale-up.

Our findings challenge the current paradigm in olive waste biorefining and offer a more economically viable and environmentally sustainable pathway for producing antioxidant-rich extracts suitable for use in cosmetics, food, and pharmaceutical applications.

## Data Availability

The original contributions presented in this study are included in the article. Further inquiries can be directed to the corresponding authors.

## Supplementary Materials

The following supporting information can be downloaded at: https://www.mdpi.com/article/10.3390/antiox14121441/s1, Table S1: List of the candidate points for the D-optima experimental plan. Table S2: MAE ten-experiment D-optimal experimental plan and three validation experiments (Exp#11–13). Table S3: UAE ten-experiment D-optimal experimental plan and three validation experiments (Exp#11–13). Table S4: mg of gallic acid per g of extract determined by Folin– Ciocâlteu assay. Table S5: μmol TE per g of extract by ORAC assay. Table S6: Confidence levels corresponding to the 95% confidence intervals for all experimental groups in DCFH-DA assay. Figure S1: Dose–response curve for extract from leaves (orange), branches (grey), and pruning waste (red) and Greenselect^®^ (light blue) determined through DPPH assay.

## Abbreviations

The following abbreviations are used in this manuscript:

% FRS	Percent Free Radical Scavenging
AAPH	2,2′-Azobis(2-methylpropionamidine) dihydrochloride
ACN	Acetonitrile
AUC	Area Under the Curve
ANOVA	Analysis of Variance
CAT	Chemometric Agile Tool
CC (cc)	Cubic Centimeter
CI	Confidence Interval
CO_2_	Carbon Dioxide
DAD/PDA	(UV-Vis) Photodiode Array Detector
DCFH–DA	2′,7′-Dichlorodihydrofluorescein Diacetate
DCM	Dichloromethane
DMEM	Dulbecco’s Modified Eagle’s Medium
DMSO	Dimethyl Sulfoxide
DoE	Design of Experiments
DW	Dry Weight
FBS	Fetal Bovine Serum
GA	Gallic Acid
H_2_O_2_	Hydrogen Peroxide
HaCaT	Human Adult Keratinocyte Cell Line
HLB	Hydrophilic–Lipophilic Balanced
HPLC	High-Performance Liquid Chromatography
ICH Q2(R2)	International Council for Harmonisation, Q2(R2)
IC_50_	Half-Maximal Inhibitory Concentration
MAE	Microwave-Assisted Solvent Extraction
MeOH	Methanol
MTT	3-(4,5-Dimethylthiazol-2-yl)-2,5-diphenyltetrazolium bromide
Na_2_CO_3_	Sodium Carbonate (anhydrous)
nm	Nanometer
OD	Optical Density
ORAC	Oxygen Radical Absorbance Capacity
PBS	Phosphate-Buffered Saline
PSI	Pound per Square Inch
QL	Quantification Limit
ROS	Reactive Oxygen Species
SD	Standard Deviation
SPE	Solid-Phase Extraction
TE	Trolox Equivalents
TPC	Total Phenolic Content
Trolox	(±)-6-Hydroxy-2,5,7,8-tetramethylchromane-2-carboxylic acid
UHPLC	Ultra-High-Performance Liquid Chromatography
UAE	Ultrasound-Assisted Extraction
UV-Vis	Ultraviolet–Visible
W	Watt
