# Supplementary material for: Unseparated Olive Pruning Waste as a Sustainable Feedstock: DoE-Optimized Extracts with Antioxidant Activity Equivalent to Isolated Leaves
Source: Antioxidants (Basel). 2025 Nov 29;14(12):1441. doi: 10.3390/antiox14121441 (PMC12729310; doi:10.3390/antiox14121441)
Supplement: Supplementary file 1 [file antioxidants-14-01441-s001.zip › antioxidants-3974840-supplementary.pdf]

**Table S1**: List of the candidate points for the D-optima experimental plan

| Exp# | $X_1$ Temperature | $X_2$ Cycles | $X_3$ Solvent Composition | $X_4$ Branches | $X_5$ Leaves |
|---|---|---|---|---|---|
| 1 | -1 | -1 | -1 | 1 | 0 |
| 2 | 1 | -1 | -1 | 1 | 0 |
| 3 | -1 | 1 | -1 | 1 | 0 |
| 4 | 1 | 1 | -1 | 1 | 0 |
| 5 | -1 | -1 | 1 | 1 | 0 |
| 6 | 1 | -1 | 1 | 1 | 0 |
| 7 | -1 | 1 | 1 | 1 | 0 |
| 8 | 1 | 1 | 1 | 1 | 0 |
| 9 | -1 | -1 | -1 | 0 | 1 |
| 10 | 1 | -1 | -1 | 0 | 1 |
| 11 | -1 | 1 | -1 | 0 | 1 |
| 12 | 1 | 1 | -1 | 0 | 1 |
| 13 | -1 | -1 | 1 | 0 | 1 |
| 14 | 1 | -1 | 1 | 0 | 1 |
| 15 | -1 | 1 | 1 | 0 | 1 |
| 16 | 1 | 1 | 1 | 0 | 1 |
| 17 | -1 | -1 | -1 | 0 | 0 |
| 18 | 1 | -1 | -1 | 0 | 0 |
| 19 | -1 | 1 | -1 | 0 | 0 |
| 20 | 1 | 1 | -1 | 0 | 0 |
| 21 | -1 | -1 | 1 | 0 | 0 |
| 22 | 1 | -1 | 1 | 0 | 0 |
| 23 | -1 | 1 | 1 | 0 | 0 |
| 24 | 1 | 1 | 1 | 0 | 0 |

**Table S2** MAE ten-experiment D-optimal experimental plan and three validation experiments (Exp#11-13).

| Exp# | $X_1$ Temperature | $X_2$ Cycles | $X_3$ Solvent Composition | $X_4$; $X_5$ matrix | $X_1$ Temperature | $X_2$ Cycles | $X_3$ Solvent Composition | $X_4$ Branches | $X_5$ Leaves | $Y_1$ Amount of oleuropein[a] (mg g$^{-1}$ DW) | $Y_2$ %FRS [b] |
|---|---|---|---|---|---|---|---|---|---|---|---|
| **1** | 90 | 1 | 20/80 | Branches | 1 | -1 | -1 | 1 | 0 | **73.2** | **53.3** |
| **2** | 40 | 3 | 20/80 | Branches | -1 | 1 | -1 | 1 | 0 | **48.9** | **47.3** |
| **3** | 40 | 1 | 80/20 | Branches | -1 | -1 | 1 | 1 | 0 | **23.0** | **68.9** |
| **4** | 90 | 3 | 80/20 | Branches | 1 | 1 | 1 | 1 | 0 | **39.5** | **44.7** |
| **5** | 90 | 1 | 20/80 | Leaves | 1 | -1 | -1 | 0 | 1 | **88.3** | **72.2** |
| **6** | 40 | 3 | 20/80 | Leaves | -1 | 1 | -1 | 0 | 1 | **76.3** | **69.3** |
| **7** | 40 | 1 | 20/80 | Pruning waste | -1 | -1 | -1 | 0 | 0 | **51.2** | **53.6** |
| **8** | 90 | 3 | 20/80 | Pruning waste | 1 | 1 | -1 | 0 | 0 | **68.8** | **56.4** |
| **9** | 90 | 1 | 80/20 | Pruning waste | 1 | -1 | 1 | 0 | 0 | **59.4** | **63.7** |
| **10** | 40 | 3 | 80/20 | Pruning waste | -1 | 1 | 1 | 0 | 0 | **57.6** | **49.2** |
| | | | | | **Validation trials** | | | | | | |
| **11** | 65 | 2 | 50/50 | Pruning waste | 0 | 0 | 0 | 0 | 0 | **68.2** | **56.1** |
| **12** | 65 | 2 | 50/50 | Pruning waste | 0 | 0 | 0 | 0 | 0 | **76.8** | **50.8** |
| **13** | 65 | 2 | 50/50 | Pruning waste | 0 | 0 | 0 | 0 | 0 | **66.0** | **50.3** |

[a] Oleuropen was quantified in the extract by UHPLC-UV/PDA through calibration curves after chlorophyll removal

[b] determined through DPPH assay at a single dose (0.4 mg mL$^{-1}$ in methanol)

**Table S3** UAE ten-experiment D-optimal experimental plan and three validation experiments (Exp#11-13).

| **Exp #** | *$X_1$* **Temperature** | *$X_2$* **Cycles** | *$X_3$* **Solvent Composition** | *$X_4$; $X_5$* **matrix** | *$X_1$* **Temperature** | *$X_2$* **Cycles** | *$X_3$* **Solvent Composition** | *$X_4$* **Branches** | *$X_5$* **Leaves** | *$Y_1$* **Amount of Oleuropein [a] (mg g$^{-1}$ DW)** | *$Y_2$* **%FRS [b]** |
|---|---|---|---|---|---|---|---|---|---|---|---|
| **1** | 80 | 1 | 20/80 | Branches | 1 | -1 | -1 | 1 | 0 | **35.3** | **47.0** |
| **2** | 30 | 3 | 20/80 | Branches | -1 | 1 | -1 | 1 | 0 | **28.0** | **51.4** |
| **3** | 30 | 1 | 80/20 | Branches | -1 | -1 | 1 | 1 | 0 | **32.1** | **53.3** |
| **4** | 80 | 3 | 80/20 | Branches | 1 | 1 | 1 | 1 | 0 | **36.6** | **46.8** |
| **5** | 80 | 1 | 20/80 | Leaves | 1 | -1 | -1 | 0 | 1 | **54.3** | **62.4** |
| **6** | 30 | 3 | 20/80 | Leaves | -1 | 1 | -1 | 0 | 1 | **61.8** | **56.3** |
| **7** | 30 | 1 | 20/80 | Pruning waste | -1 | -1 | -1 | 0 | 0 | **29.6** | **51.6** |
| **8** | 80 | 3 | 20/80 | Pruning waste | 1 | 1 | -1 | 0 | 0 | **59.6** | **54.8** |
| **9** | 80 | 1 | 80/20 | Pruning waste | 1 | -1 | 1 | 0 | 0 | **62.7** | **61.0** |
| **10** | 30 | 3 | 80/20 | Pruning waste | -1 | 1 | 1 | 0 | 0 | **69.0** | **56.0** |
| | | | | | **Validation trials** | | | | | | |
| **11** | 55 | 2 | 50/50 | Pruning waste | 0 | 0 | 0 | 0 | 0 | **45.6** | **55.8** |
| **12** | 55 | 2 | 50/50 | Pruning waste | 0 | 0 | 0 | 0 | 0 | **65.6** | **54.9** |
| **13** | 55 | 2 | 50/50 | Pruning waste | 0 | 0 | 0 | 0 | 0 | **70.9** | **50.0** |

[a] Oleuropen was quantified in the extract by UHPLC-UV/PDA through calibration curves after chlorophyll removal

[b] determined through DPPH assay at a single dose (0.4 mg mL$^{-1}$ in methanol)

**Table S4.** mg of Gallic acid per g of extract determined by Folin– Ciocâlteu assay.

| mg of Gallic acid /g extract* | | |
|---|---|---|
| **Branches** | **Leaves** | **Pruning waste** |
| 148.41±0.001 | 192.51 ± 59.85 | 186.60 ±16.21 |

*Results are reported as mean ± SD of three experiments

**Table S5.** μmol TE per g of extract by ORAC assay.

| μmol TE/g of extract* | | |
|---|---|---|
| **Branches** | **Leaves** | **Pruning waste** |
| 46.98 ± 9.84 | 103.78 ± 9.88 | 99.95 ± 16.98 |

*Results are reported as mean ± SD of three experiments

**Table S6** Confidence levels corresponding to the 95% confidence intervals for all experimental groups in DCFH-DA assay.

| **Conditions** | **Basal** | | | | **$H_2O_2$** | | | |
|---|---|---|---|---|---|---|---|---|
| Group | Control | Leaves | Branches | Pruning waste | Control | Leaves | Branches | Pruning waste |
| Confidence levels | 20.30 | 31.08 | 25.64 | 12.79 | 113.26 | 51.28 | 23.87 | 91.01 |

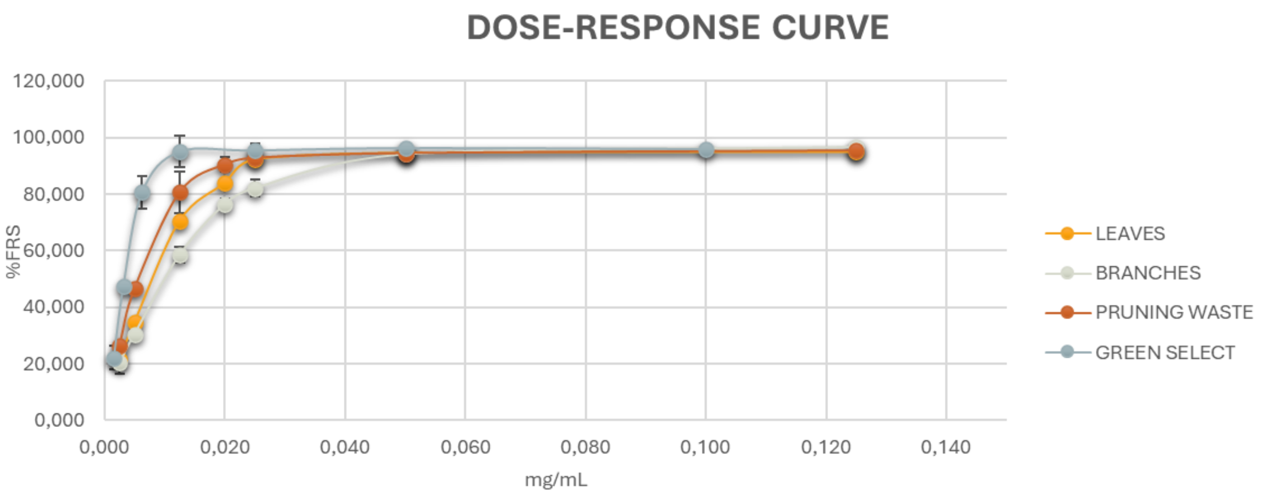


**Figure S1**: Dose-response curve for extract from leaves (orange) ,branches (grey) and pruning waste ( red) and Greenselect® (light blue) determined through DPPH assay.
